# Assessment of Continuous Epidural Analgesia Versus Continuous Surgical Transverse Abdominis Plane Block for Postoperative Analgesia in Gynecological Surgeries

**DOI:** 10.7759/cureus.49957

**Published:** 2023-12-05

**Authors:** Lakshmipriya Ilangovan, Sivaperumal G, Sathyasuba Meenakshisundaram, Karthikeyan Selvaraj, Raghuraman M Sethuraman, Iswaryarajan Hercule M.S, Hiremath P.B.

**Affiliations:** 1 Anesthesiology, Melmaruvathur Adhiparasakthi Institute of Medical Sciences, Chennai, IND; 2 Anesthesiology, Aarupadai Veedu Medical College, Puducherry, IND; 3 Anesthesiology, Sree Balaji Medical College and Hospital, Bharath Institute of Higher Education and Research (BIHER), Chennai, IND; 4 General Surgery, Sree Balaji Medical College and Hospital, Bharath Institute of Higher Education and Research (BIHER), Chennai, IND; 5 Anesthesiology, Sri Venkateswaraa Medical College and Hospital, Puducherry, IND; 6 Obstetrics and Gynecology, Sri Venkateswaraa Medical College and Hospital, Puducherry, IND

**Keywords:** continuous wound infusion, open gynecological operations, postoperatve analgesia, epidural analgesia, surgical transverse abdominis plane block

## Abstract

Background

Surgical transverse abdominis plane (TAP) block has been studied in various surgeries. However, its role particularly in the form of continuous infusion in comparison to epidural infusion in open gynecological surgeries remains unknown. Hence, this study was taken up.

Methodology

Sixty patients were assigned to either of the two groups: continuous epidural (Group E) or continuous infusion in the surgical TAP (Group S). The primary outcomes were visual analog scale (VAS) pain scores and rescue analgesic requirements. Postoperative complications such as nausea/vomiting, hypotension, and bradycardia were also assessed.

Results

Mean pain scores were significantly lesser in Group E. However, 80% (24) of Group E and 50% (15) of Group S did not require rescue analgesia, which was not statistically significant. Adverse effects did not differ significantly between the two groups.

Conclusions

The efficacy of the continuous surgical TAP block is similar to a continuous epidural. Therefore, it can be considered in settings with limited resources and expertise or in cases of contraindications for an epidural.

## Introduction

Control of postoperative pain is very important for providing patient comfort, early mobilization, and fast recovery, to minimize the neuroendocrine stress response and postoperative organ dysfunction [1]. Epidural analgesia is an effective technique that is widely used in the management of postoperative pain. It reduces postoperative pulmonary complications, minimizes surgical stress responses, and provides early ambulation and restoration of bowel function and health-related quality of life [2].

*Surgical TAP* (transverse abdominis plane) block [3] is a simple, safe, and easy technique, wherein a catheter is placed between the transverse abdominis and internal oblique muscles by the surgeon during the closure of the wound and local anesthetic is administered as a continuous infusion through this catheter. A few studies have analyzed the effectiveness of postoperative pain relief between epidural analgesia and surgical TAP infusion in open nephrectomies or other renal surgeries [4-6]. Another variant of this technique called *surgeon-placed TAP block* [7], in which the surgeon performs a single injection, has also been analyzed in a few studies [7-9]. However, to our knowledge, no prospective, randomized study has compared the effectiveness of continuous epidural analgesia and continuous infusion of surgical TAP block in open gynecological surgeries. Although ultrasound-guided TAP block has emerged as a promising technique recently, its availability, expertise, and time are major prohibiting factors. A catheter technique further compounds this shortcoming. Hence, we took this study to assess the role of surgical TAP block in this population. We hypothesized that surgical TAP block would be as effective as epidural analgesia in open gynecological surgeries.

## Materials and methods

This randomized, double-blind study was conducted at a tertiary care teaching hospital over 15 months after approval from the institutional ethics committee, and the study was registered prospectively with the clinical trial registry of India (CTRI/2019/01/017057). Patients of the American Society of Anesthesiologists (ASA) physical status I or II posted for gynecological surgeries under spinal anesthesia were included, and written informed consent was obtained from the participants. Patients with a coagulation disorder, the presence of infection at the surgical site, psychiatric disorders, allergies to local anesthetics and diclofenac sodium, and patients converted to general anesthesia were excluded.

A total of 60 patients were divided into two groups (Group E or S) by lottery method. After completing a comprehensive pre-anesthetic checkup, the patients were informed about the analgesic method to be used after surgery and were given details about the postoperative pain questionnaires. All patients received premedication with pantoprazole 40 mg, metoclopramide 10 mg, and alprazolam 0.5 mg orally the night before surgery. On the day of surgery, after shifting the patient inside the operation theater, standard monitors were connected, an intravenous line was secured, and a lactated Ringer's solution was started.

In Group E, an epidural catheter was inserted before spinal anesthesia. The procedure was performed in the sitting position. We identified the lumbar epidural space at L1 -L2 or L2 -L3 by the loss of resistance technique using an 18-G Tuohy needle. After identifying the space, a multi-orifice 20 G epidural catheter was placed 3 to 4 cm within the epidural space. A test dose of 3 mL of 2% lidocaine with epinephrine (5 mcg/mL) was given through the catheter.

Both the groups received spinal anesthesia with 3.5 mL of 0.5% hyperbaric bupivacaine in the sitting position at L3-L4 or L4 -L5 space, depending upon anatomical factors of the individual patient and the space used for epidural catheter insertion in Group E.

In Group S, at the end of the operation, the surgeon identified the transversus abdominis muscle at the upper and lower ends. The muscle was grasped by an Allis clamp, and a plane was developed between the transversus abdominis and the internal oblique muscle using both blunt and sharp dissection. At the end of the dissection, a small mosquito clamp was passed through the wound layers with a skin exit of 2 cm from the upper end of the incision, and an infant feeding tube, size 8, with multiple holes was placed in the dissected plane. The catheter was fixed to the skin, and the abdomen was closed in layers.

Patients were transferred to the postoperative care area for observation. Once the sensory level of spinal anesthesia receded to T10, Group E received epidural analgesia with 5 mL of 0.2% ropivacaine as a bolus. This dose and concentration were adhered to in accordance with institutional practice as it produces only sensory and no motor block. It was followed by 0.2% ropivacaine at the rate of 5 mL/hour as a continuous infusion for 36 hours. Group S also received the same bolus and infusion through the wound catheter for 36 hours. Both the epidural and the surgical TAP catheters were removed at the end of 36 hours of the postoperative period. Injection diclofenac sodium 75 mg was administered intramuscularly if the pain score was more than 4 as a rescue analgesic.

The VAS pain scores and rescue analgesic requirements were the primary outcomes. Postoperative complications such as nausea/vomiting, hypotension, and bradycardia were also assessed as secondary outcomes. Postoperatively, these parameters were assessed by the anesthesiologist not involved in the study: (1) Pain was assessed using VAS 10 cm unmarked line, with 0 cm = no pain and 10 cm = worst pain, at 0, 5, and 30 minutes and at 1, 6, 12, 24, and 36 hours postoperatively. (2) Number of rescue analgesia required in 36 hours. (3) The incidence of postoperative complications such as nausea/vomiting, hypotension (mean arterial blood pressure ≤60 mmHg), and bradycardia (heart rate <60 minute^-1^).

Sample size calculation

We calculated the sample size following a pilot study. The mean pain score of CWI patients was 0.8, while it was 1.15 for the epidural technique. Considering the power at 80% with 95% confidence interval, we arrived at a sample size of 60 with a 1:1 split-up of 30 in each group.

Statistical analysis

The collected data were analyzed using IBM SPSS Statistics for Windows, Version 26.0 (IBM Corp., Armonk, NY). Descriptive statistics frequency analysis and percentage analysis were used for categorical variables, and the mean and standard deviation were used for continuous variables. To find the significant difference between the bivariate samples in independent groups, the unpaired sample t-test and the Mann-Whitney U test were used. To find the significance in categorical data, the chi-square test was used. If the expected cell frequency was less than 5 in 2 × 2 tables, the Fisher's Exact test was used. In all the aforementioned statistical tools, the *P*-value of 0.05 was considered significant.

## Results

This study was conducted on a total of 60 patients randomized into two groups. The age distribution among the 60 patients was as follows: three patients in the 26- to 35-year age group for Group E compared to two patients for Group S, 13 patients in the 36- to 45-year age group for Group E compared to 18 patients for Group S, and 14 patients in the 46- to 55-year age group for Group E compared to 10 patients for Group S (*P* = 0.433). There was an equal distribution of ASA class I (20 each) and ASA class II (10 each) patients in the two groups. The performed surgeries are as follows: total abdominal hysterectomy with bilateral salpingo-oophorectomy (22 cases in Group E, 19 in Group S); total abdominal hysterectomy (three cases in Group E, five in Group S); ovarian cystectomy (five cases in Group E, six in Group S). The duration of surgeries between the two groups was comparable, with a mean of 108 minutes for Group E and 101.6 minutes for Group S (*P* = 0.26).

The mean pain scores at rest for 36 hours were significantly lower in Group E compared to Group S (Table 1). However, 80% of Group E and 50% of Group S patients did not require any rescue analgesia, which was not statistically significant (Table 2). Only two patients of Group S vomited, while none in Group E (*P *= 0.15). Also, other adverse effects did not differ significantly between the two groups. All patients were ambulant by the second postoperative day, and the urinary catheter, which was put after spinal anesthesia administration, was also removed on the second postoperative day. None of the patients had urinary retention after catheter removal.

**Table 1:** Comparison of pain scores between groups. The range of pain score was 0-6 for Group E and 2-8 for Group S. The Mann-Whitney U test was used to find out *P*- and *Z*-values. ^#^No statistical significance at *P* > 0.05. ^*^Statistical significance at *P* < 0.05. ^**^Highly significant at *P* < 0.01. SD, standard deviation

Duration	Groups	n	Mean	SD	*Z*-value	*P*-value
0 minute	Group E	30	4.7	1.2	1.234	0.217^#^
	Group S	30	4.3	1.5		
30 minutes	Group E	30	2.9	1.1	2.423	0.015^*^
	Group S	30	3.9	1.5		
1st hour	Group E	30	2.1	0.8	4.592	0.0005^**^
	Group S	30	3.8	1.6		
6th hour	Group E	30	2.5	1.5	3.315	0.001^**^
	Group S	30	3.7	1.4		
12th hour	Group E	30	2.6	1.3	3.266	0.001^**^
	Group S	30	3.5	1.1		
24th hour	Group E	30	1.9	1.2	2.935	0.003^**^
	Group S	30	2.7	1.1		
36th hour	Group E	30	1.1	1.0	2.574	0.010^*^
	Group S	30	1.9	1.0		

**Table 2:** Comparison of rescue analgesics requirements. The chi-square test was used.

	Groups		Total, *n* (%)	*X*^2^-value	*P*-value
	Group E, *n* (%)	Group S, *n* (%)			
Number of rescue analgesics					
I	5 (16.7)	11 (36.7)	16 (26.7)	6.327	0.097
II	1 (3.3)	3 (10)	4 (6.7)		
III	0 (0)	1 (3.3)	1 (1.7)		
Nil	24 (80)	15 (50)	39 (65)		
Total	30 (100)	30 (100)	60 (100)		

## Discussion

In this study, we observed continuous epidural infusion (CEI) significantly reduced pain scores when compared to surgical TAP infusion although the requirements of rescue analgesics were comparable. Multimodal analgesia with minimal or no opioids is gaining popularity to provide good-quality analgesia with lesser side effects. In accordance with our study, Bertoglio et al. [1] also observed no significant difference between preperitoneal continuous wound infusion (CWI) and CEI groups regarding pain scores and rescue analgesics in open colorectal cancer surgeries. While we monitored the pain scores at rest only for 36 hours, Bertoglio et al. [1] continued it until 72 hours, besides observing pain scores at coughing. The return of bowel function was significantly earlier in the CWI group in their study [1].

The CWI through surgical TAP was also analyzed in open nephrectomy patients [4,5]. Forastiere et al. concluded that the CWI of ropivacaine compared to the CWI of saline produced better postoperative pain relief, early recovery, and discharge, resulting in cost reduction [4]. In contrast, Capdevila et al. [5] compared CWI with CEI and a control group receiving patient-controlled morphine administration. The pain scores and morphine consumption were significantly lower in the CWI and CEI groups. They concluded that CWI and CEI significantly improved postoperative analgesia, reduced the area of wound hyperalgesia, and accelerated the rehabilitation process. In addition, CWI significantly reduced the magnitude of residual pain at one month and optimizes quality-of-life parameters at three months follow-up. It is important to note that an additional catheter in the subcutaneous space was used in these two studies [4,5]. In contrast to these two studies, Heba et al. [6] used a single catheter for CWI of surgical TAP in open renal surgeries with flank incisions and observed its effectiveness to be equal to CEI. The results of that study were similar to our study. However, the incidences of hypotension and motor block, which delayed ambulation, were higher in the epidural group [6].

As mentioned in the Introduction section, *surgeon-placed TAP block* [7] has also been analyzed in a few studies [7-9]. For instance, Kay et al. [7] performed a retrospective analysis in which they compared a single injection of surgeon-placed TAP block with a thoracic epidural for gynecological oncosurgeries. Their study highlighted the advantages of surgeon-placed TAP in terms of lesser time spent in the operating room before the commencement of surgery, thereby incurring decreased costs to the patient, use of opioid-sparing medications, and adherence to enhanced recovery after surgery protocol, tiding over situations of non-availability of ultrasound, etc. At the same time, they had concluded that this was not generalizable to all surgical patients, and patients with upper abdominal incisions and chronic pain would probably benefit from an epidural. Owen et al. [8] also performed the same technique in cesarean section patients and found that it was easier, safer, and equally effective when compared to the conventional TAP method. Similarly, Urfalıoğlu et al. [9] compared a single-dose surgical TAP with ultrasound-guided TAP and observed that surgical TAP was technically simpler and less time-consuming than the ultrasound-guided method and that both techniques were similar in efficacy and safety in obese cesarean section patients.

Ayad et al. [10] compared a single-shot TAP infiltration of liposomal bupivacaine, CEI with patient-controlled intravenous analgesia. They observed that TAP infiltration and epidural were similar with regard to pain and opioid consumption. They concluded that TAP infiltrations may be considered an alternative to epidural analgesia in abdominal surgeries. Their study compared only the somatic pain relief in both groups because they suggested that somatic pain contributed the most to abdominal surgeries. However, we did not assess somatic and visceral pain individually, which could be the reason that surgical TAP had higher VAS scores when compared to that of CEI.

Jouve et al. [11] observed that CEI was better than CWI through a catheter placed between the parietal peritoneum and transversalis fascia in open colorectal procedures. Tilleul et al. [12] concluded that CWI was cost-effective (more effective and less costly) when compared to patient-controlled infusion, while CWI was less costly with almost equal efficacy when compared to CEI in laparotomies.

According to a recent review article, the surgeon-administered (laparoscopic-guided) TAP block was comparable to the anesthesiologist-administered (ultrasound-guided) TAP block, with no discernible difference in patient outcomes [13]. Wong et al. in their study concluded that laparoscopic TAP blocks are safe, effective, and noninferior to ultrasound-guided TAP blocks in the immediate postoperative period [14]. Ladanyi et al. in their review article found that surgeon-performed TAP block under laparoscopic visualization is a safe and efficacious intervention to reduce postoperative pain and may add to a multimodal approach for enhanced recovery protocols [15]. Thus, laparoscopic TAP block is emerging as an alternative promising technique. Also, robot-assisted surgeries have gained popularity in recent years. In this regard, a recent retrospective study by Shahait et al. was a breakthrough in concluding that robot-assisted transperitoneal TAP block is a safer alternative compared to local anesthetic port-site infiltration, with lower postoperative pain scores and less narcotic use in patients undergoing robot-assisted radical prostatectomy [16].

Our study has some limitations: (1) We did not include a control group as it was objected to by the ethical committee. If we had included the control group in our study, we might have gotten a result of surgical TAP being better than the control group. (2) Although the anesthesiologist involved in the assessment of parameters was blinded to the technique, there was still a possibility of violation because of the nature of the techniques.

## Conclusions

Although CEI significantly reduces the pain scores, the requirement of postoperative rescue analgesics was not affected significantly in our study. Surgical TAP block can be considered for postoperative pain management in clinical settings where resources and expertise are unavailable for managing CEI or in cases of its contraindication, especially in situations like patients taken up for emergency procedures where antiplatelet/antithrombotic drugs could not be withheld.
